# Organ specific responses to first-line lenvatinib plus anti-PD-1 antibodies in patients with unresectable hepatocellular carcinoma: a retrospective analysis

**DOI:** 10.1186/s40364-021-00274-z

**Published:** 2021-03-20

**Authors:** Cheng Huang, Xiao-Dong Zhu, Ying-Hao Shen, Dong Wu, Yuan Ji, Ning-Ling Ge, Ling-Li Chen, Chang-Jun Tan, Jian Zhou, Jia Fan, Hui-Chuan Sun

**Affiliations:** 1 Department of Liver Surgery and Transplantation, Liver Cancer Institute and Zhongshan Hospital, Fudan University, 180 Fenglin Road, Shanghai, 200032 China; 2 Department of Radiology, Zhongshan Hospital, Fudan University, Shanghai, 200032 China; 3 Department of Pathology, Zhongshan Hospital, Fudan University, Shanghai, 200032 China; 4 Department of Hepatic Oncology, Liver Cancer Institute and Zhongshan Hospital, Fudan University, Shanghai, 200032 China

**Keywords:** Lenvatinib, Carcinoma, Hepatocellular, Liver neoplasms, Immunotherapy

## Abstract

**Background:**

We evaluated organ-specific response rates (OSRRs) to first-line lenvatinib plus anti-PD-1 antibodies in patients with advanced hepatocellular carcinoma (HCC).

**Methods:**

This retrospective analysis included Chinese patients with unresectable/advanced HCC who received first-line lenvatinib (8 mg/day) plus ≥3 infusions of anti-PD-1 antibodies between October 2018 and May 2020. Tumor and macrovascular tumor thrombi (MVTT) treatment responses were evaluated every 2 months using RECIST v1.1. The overall response rate (ORR)/OSRR was defined as the percentage of patients with a best overall response of complete or partial response (CR or PR).

**Results:**

In total, 60 patients were included in the analysis; 96.7% had measurable intrahepatic lesions, 55% had MVTT and 26.7% had extrahepatic disease. In all 60 patients, the ORR was 33.3%, median progression-free survival was 7.0 months (95% CI, 1.7–12.3) and median overall survival was not reached. The OSRR for MVTT (54.5%) was higher versus intrahepatic tumors (32.8%), extrahepatic lung metastases (37.5%) and lymph node metastases (33.3%). Among 33 patients with intrahepatic tumors and MVTT, 18 had differential responses in each site, including 13 with a better response in MVTT versus intrahepatic lesions. Among 18 patients whose MVTT achieved a radiographic CR or PR, six underwent surgical resection: 4/6 achieved a pathological CR in MVTT and 2/6 in the intrahepatic tumor.

**Conclusions:**

First-line lenvatinib plus anti-PD-1 antibodies resulted in better tumor responses in MVTT versus intrahepatic lesions. Complete MVTT necrosis may allow downstaging and subsequent eligibility for surgical resection in a proportion of patients with advanced HCC.

## Introduction

Hepatocellular carcinoma (HCC) is the most common cancer of the liver, and is the fourth-most-common cause of cancer-related death worldwide [1]. Patients with early and intermediate stage HCC can access treatment options associated with the best long-term survival, including liver resection, orthotopic liver transplant and locoregional therapies [2]. However, for patients diagnosed with advanced HCC (accounting for > 50% of patients [3]) or those who progress following locoregional therapy, treatment options are usually limited to systemic therapy and the prognosis is often poor [4, 5]. The past decade has seen significant advances in systemic therapy for advanced HCC. Sorafenib was approved in this indication in 2008, [6] and lenvatinib was approved in 2018 after showing non-inferiority to sorafenib in the REFLECT trial [7, 8]. More recently, immunotherapy with anti-programmed cell death-1 (PD-1) antibodies (nivolumab and pembrolizumab) has been investigated in advanced HCC, with mixed results [9–11].

Mechanistic research supports a synergistic effect of combined treatment with immune therapy and VEGF inhibitors, and several such combination therapies have been investigated in HCC [12]. Combined therapy with atezolizumab (an anti-programmed cell death ligand-1 antibody) plus bevacizumab has been approved for the treatment of advanced HCC after showing superior overall survival (OS) and progression-free survival (PFS) to sorafenib in the Phase III IMbrave150 trial [13]. Combination therapy with lenvatinib and anti-PD-1 antibodies has also shown promising early results in patients with unresectable HCC (objective response rate [ORR] = 36.0%; median OS = 22 months) [14]. These outcomes compare favorably to those reported for lenvatinib monotherapy in the REFLECT trial (ORR = 24.1%; median OS = 13.6 months), [8] and to single-agent nivolumab (ORR = 15%; median OS = 16.4 months) and pembrolizumab (median OS = 13.9 months) in advanced HCC [10, 11].

A large proportion of patients with HCC are diagnosed at a late stage with metastatic disease [15]. Differential responses to treatment have been observed for HCC lesions in different sites and organs, which has been attributed to the heterogeneity of the tumor immune microenvironment across different organs [16, 17]. Gaining more understanding of the heterogeneous treatment responses of HCC tumors in different anatomical sites would support the development of new treatment strategies, with the potential to prolong patient survival.

Here, we report a retrospective analysis of the response of HCC lesions in different organs (organ-specific response rate [OSRR]) to first-line treatment with combined lenvatinib and anti-PD-1 antibodies. In particular, we evaluate treatment response in macrovascular tumor thrombi (MVTT), which is a common characteristic of patients with advanced liver cancer. To our knowledge, this represents the first report of OSRR to first-line treatment of HCC and the first report of OSRR following combination treatment with lenvatinib and anti-PD-1 antibodies.

## Methods

### Aim

This study aimed to investigate differential responses of HCC lesions in different organs to first-line treatment with combined lenvatinib and anti-PD-1 antibodies.

### Study design and patient population

This was a retrospective analysis of data from patients with unresectable or advanced HCC who received first-line treatment with lenvatinib plus ≥3 infusions of anti-PD-1 monoclonal antibodies at Zhongshan Hospital, Fudan University, China between October 2018 and May 2020. HCC diagnosis was based on tissue histology, or clinically confirmed according to the American Association for the Study of Liver Diseases criteria [18]. The presence and extent of vascular invasion was evaluated based on contrast-enhanced dynamic MRI findings. No patients received anti-coagulation therapy for vascular tumor thrombi prior to enrolment, and no patients received other systemic anti-cancer therapies before entering the study.

Prior to treatment administration, all patients underwent a baseline evaluation that included liver, renal, thyroid, adrenal and cardiac function tests, complete blood count, and testing for hepatitis B surface antigen (HBsAg), HBV DNA, alpha-fetoprotein (AFP) and protein induced by vitamin K absence-II (PIVKA-II). All patients received ≥3 infusions of anti-PD-1 antibodies and completed ≥1 efficacy and safety assessment.

All patients were monitored regularly, including repeat safety evaluations 2–3 days prior to each anti-PD-1 antibody treatment cycle. The study protocol, including treatment regimen and data collection, were approved by Zhongshan Hospital Research Ethics Committee, and written informed consent was obtained from all patients before initiation of treatment.

### Systemic therapy

All patients received lenvatinib (8 mg/day regardless of body weight; Eisai, Inc., Japan) plus anti-PD-1 antibodies. Anti-PD-1 antibodies were intravenously administered as follows: nivolumab (Bristol-Myers Squibb, USA) 3 mg/kg or camrelizumab (Hengrui Medicine, China) 200 mg, [19] every 2 weeks, or pembrolizumab (MSD, USA) 200 mg, sintilimab (Innovent Biologics, China) 200 mg, [20] or toripalimab (Junshi Bioscience, China) 240 mg, [21] every 3 weeks. All patients with active hepatitis B infection received concomitant anti-viral therapy.

### Response evaluation

Tumor responses were evaluated using abdominal contrast-enhanced MRI and chest serial CT every 2 months (±2 weeks), and the Response Evaluation Criteria in Solid Tumors (RECIST) v1.1 was used to evaluate overall ORR and OSRR, and duration of response (DOR) [22]. All response evaluations were subject to investigator (INV) and independent imaging review (IIR) groups’ assessments.

Response of MVTT (in the portal veins, hepatic veins or vena cava) to combined therapy was evaluated by contrast-enhanced MRI, and the product of the largest perpendicular diameters of the tumor thrombus was calculated and compared to the initial value, irrespective of the vascular site. The maximum perpendicular diameter of a tumor thrombus ≥1.0 cm was considered to be a measurable lesion. A complete response (CR) was defined as the complete disappearance of the MVTT, partial response (PR) as a ≥ 30% decrease in the thrombus diameter, stable disease (SD) as between a 30% decrease and a 20% increase in thrombus diameter, and progressive disease (PD) as ≥20% increase in thrombus diameter [23].

The ORR was defined as the percentage of patients with a best overall response of CR or PR. The OSRR was defined as the percentage of patients with CR or PR as the best response in the MVTT or specific organs. The disease control rate (DCR) was defined as the percentage of patients who achieved CR, PR or SD as the best overall response. The organ specific DCR (OSDCR) was defined as the percentage of patients who achieved CR, PR, or SD as the best organ-specific response.

### Statistical analysis

Statistical analyses were performed using PASW Statistics v.18.0 for Windows (IBM Corp., Armonk, NY, USA). Results were summarized using descriptive statistics; continuous variables were summarized as mean (standard deviation) or median (range) unless specified, and binary variables were summarized as n (%). ORR and DCR were calculated with 95% CI using the Clopper-Pearson method (https://epitools.ausvet.com.au/). OS was defined as the interval between the date that combination therapy was initiated and the date of the patient’s death. PFS was defined as the interval between initiation of combination therapy and disease progression or death. DOR, PFS and OS were estimated using the Kaplan-Meier method and the log-rank test was used to compare survival for patients who did and did not respond to therapy. All statistical tests were two-sided and *P* < 0.05 was considered statistically significant.

## Results

### Baseline characteristics

Overall, 60 patients met the eligibility criteria and were included in the analysis. Patient demographics and baseline disease characteristics are shown in Table 1. In brief, patients were predominantly male (*n* = 55) with a mean age of 54.0 ± 10.3 years, and 40% had an Eastern Cooperative Oncology Group (ECOG) performance status score of 0. The majority of patients were positive for HBsAg (85.0%), were Child-Pugh Class A (96.7%) and had Barcelona Clinic Liver Cancer (BCLC) Stage C disease (76.7%). Half of the patients had China National Liver Cancer (CNLC) Stage IIIa disease (with MVTT and without extrahepatic metastasis), [24] while approximately one quarter (26.7%) were classified as CNLC Stage IIIb (with extra-hepatic metastasis). Of the 30 patients with CNLC Stage IIIa, three had unevaluable MVTT (the diameter was < 1.0 cm). Of the 16 patients with CNLC Stage IIIb, six had MVTT and all were evaluable.

**Table 1:** Patient demographics and baseline characteristics

	Patients (***N*** = 60)
**Mean age, years ± SD**	54.0 ± 10.3
**Sex,** ***n*** **(%)**	
Male	55 (91.7)
Female	5 (8.3)
**ECOG PS score,** ***n*** **(%)**	
0	24 (40.0)
≥ 1	36 (60.0)
**HBsAg,** ***n*** **(%)**	
Positive	51 (85.0)
Negative	9 (15.0)
**Child-Pugh class,** ***n*** **(%)**	
A	58 (96.7)
B	2 (3.3)
**α-Fetoprotein,** ***n*** **(%)**	
< 400 ng/ml	21 (35.0)
≥ 400 ng/ml	39 (65.0)
**BCLC stage,** ***n*** **(%)**	
A	2 (3.3)
B	12 (20.0)
C	46 (76.7)
**CNLC stage,** ***n*** **(%)**	
Ib	2 (3.3)
IIa	2 (3.3)
IIb	10 (16.7)
IIIa	30 (50.0)
IIIb	16 (26.7)
**Macrovascular invasion,** ***n*** **(%)**^**a**^	
Portal vein	28 (46.7)
Hepatic vein and/or vena cava	5 (8.3)
**Extrahepatic metastases,** ***n*** **(%)**	
Lung	7 (11.7)
Lymph node	6 (10.0)
Adrenal gland	2 (3.3)
Intra-abdominal implantation	2 (3.3)

*BCLC* Barcelona Clinic Liver Cancer stage, *CNLC* China Liver Cancer stage, *ECOG* Eastern Cooperative Oncology Group, *HBsAg* hepatitis B virus surface antigen

^**a**^Measurable macrovascular invasion

Of the 60 patients in the analysis, 58 had measurable intrahepatic lesions (the other two had no intrahepatic tumors; one case of abdominal implantation, and one case of abdominal lymph node metastasis). Extrahepatic lesions were present in 26.7% of patients, with two patients having extrahepatic lesions at > 1 site. There were 33 cases of MVTT, seven cases of lung metastasis, six cases of abdominal lymph node metastasis, two cases of adrenal gland metastasis and two cases of abdominal implant. These lesions were evaluated for OSRR.

### Treatments and overall response rates

**Fig. 1:**
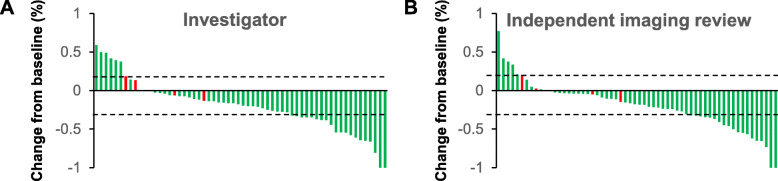
Objective responses of target lesions following treatment with lenvatinib plus anti-PD-1 antibodies. **a** Overall change from baseline in tumor size according to INV assessment. **b** Overall change from baseline in tumor size according to IIR assessment. Each bar represents one patient. Red bars represent patients whose responses were classified as PD due to the appearance of new lesions

Among all 60 patients in the analysis, the median time to discontinuation of treatment was 7.5 months (range 2–22). At the time of final follow-up (August, 2020), 35 patients remained on anti-PD-1 antibody treatment. The median time-to-response was 2.0 months (range 2–6). In the assessment by INV, CR, PR, SD and PD was achieved by two (3.3%), 18 (30.0%), 30 (50.0%), and 10 (16.7%) patients, respectively; While in the IIR assessment, CR, PR, SD and PD was achieved by two (3.3%), 18 (30.0%), 31 (51.7%), and nine (15.0%) patients. The ORR was 33.3% by both INV and IIR assessments (Fig. 1). The DCR also showed good agreement between INV and IIR assessments (83.3 vs 85.0%, respectively).

### Organ-specific response rates

Analysis of tumor response by organ showed that the OSRR in MVTT (54.5% INV, 51.5% IIR) was higher than the OSRR in intrahepatic tumors (32.8% INV & IIR), extrahepatic metastases in the lung (37.5% INV & IIR) and lymph nodes (33.3% INV & IIR). The OSRR for adrenal gland metastasis and intra-abdominal implants (both *n* = 2) was 100%, although the patient numbers were very small. A higher CR and PR rate was observed for MVTT compared with intrahepatic tumors (*P* = 0.042) (Table 2).

**Table 2:** Organ specific response rate in intrahepatic tumors and MVTT according to RECIST v1.1

Response	INV			IIR		
	Intrahepatic (***n*** = 58)	MVTT (***n*** = 33)	***P***	Intrahepatic (***n*** = 58)	MVTT (***n*** = 33)	***P***
**ORR, % (95% CI)**	32.8 (21.0–46.3)	54.5 (36.4–71.9)	0.042	32.8 (21.0–46.3)	51.5 (33.5–69.2)	0.079
**OSDCR, % (95% CI)**	84.5 (72.6–2.7)	87.9 (71.8–96.6)	0.763	86.2 (74.6–93.9)	93.9 (79.8–99.3)	0.318
**CR, % (*n/N*)**	3.4 (2/58)	9.1 (3/33)	–	3.4 (2/58)	12.1 (4/33)	–
**PR, % (*n/N*)**	29.3 (17/58)	45.5 (15/33)	–	29.3 (17/58)	39.4 (13/33)	–
**SD, % (*n/N*)**	51.7 (30/58)	33.3 (11/33)	–	54.5 (31/58)	42.4 (14/33)	–
**PD, % (*n/N*)**	15.5 (9/58)	12.1 (4/33)	–	13.8 (8/58)	6.1 (2/33)	–

*CR* complete response, *IIR* independent imaging review, *INV* investigator, *MVTT* macrovascular tumor thrombi, *ORR* objective response rate, *OSDCR* organ-specific disease control rate, *PD* progressive disease, *PR* partial response, *SD* stable disease

**Fig. 2:**
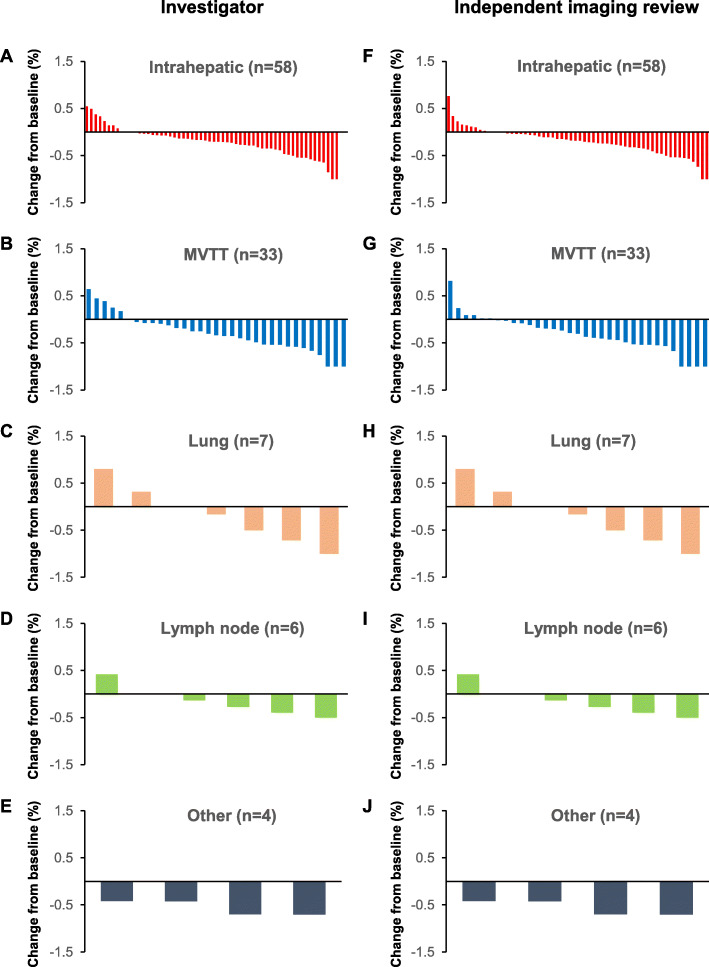
Tumor response in different organs following treatment with lenvatinib plus anti-PD-1 antibodies. **a**–**e** Change from baseline in tumor size according to INV assessment; **f**–**j** change from baseline in tumor size according to IIR assessment. Each bar represents one patient. MVTT, macrovascular tumor thrombi

According to INV assessment, the majority of patients (46/58; 79.3%) achieved a reduction in intrahepatic tumor size from baseline following treatment with lenvatinib plus anti-PD-1 antibodies. However, four patients (4/58; 6.9%) had no change in intrahepatic tumor size and eight patients (8/58; 13.8%) experienced an increase in intrahepatic tumor size following treatment (Fig. 2a). Among the 33 patients with MVTT, 81.8% (27/33) achieved a reduction in MVTT size, 15.0% (5/33) experienced an increase in MVTT size and 3.0% (1/33) experienced no change (Fig. 2b).

**Fig. 3:**
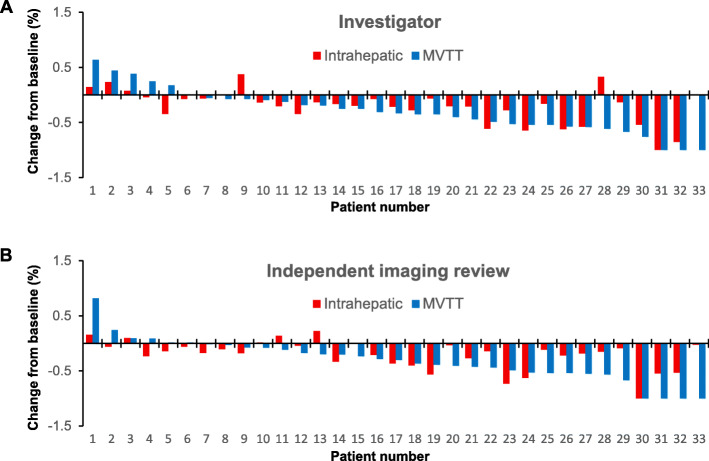
Paired intrahepatic tumor and MVTT responses following treatment with lenvatinib plus anti-PD-1 antibodies. **a** INV assessment; **b** IIR assessment. MVTT, macrovascular tumor thrombi

**Fig. 4:**
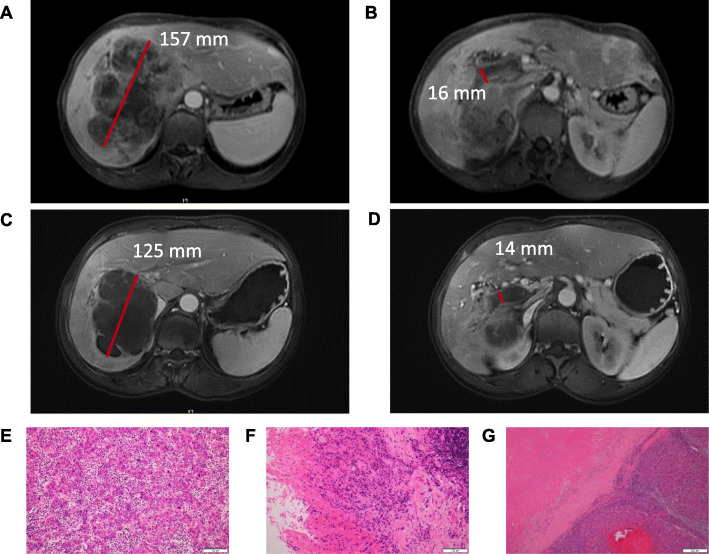
Representative sequential MRI images for one patient. The images show pre-treatment liver tumor, **a** and macrovascular tumor thrombi **b** as well as post-systemic therapy liver tumor **c** and macrovascular tumor thrombi **d**. H&E staining of resected tumor samples revealed a residual HCC area accounting for around 40% of tumor volume, and infiltration of a large number of inflammatory cells into the interstitium, ×200 **e**, complete necrosis of the macrovascular tumor thrombi with massive inflammatory cell infiltration and areas of necrosis, × 200 **f** and residual hepatocellular carcinoma, ×40 **g**

By INV evaluation, among 33 patients with both intrahepatic tumor and MVTT, a total of 18 had differential responses for intrahepatic tumor versus MVTT, of whom 13 achieved a better response in the tumor thrombus versus the intrahepatic tumor (Fig. 3a and b). Among 18 patients whose MVTT achieved a radiographic CR or PR according to INV assessment, six underwent surgical resection. Of these six patients, 4/6 achieved a pathological complete response (pCR) in MVTT and 2/6 in the intrahepatic tumor (Fig. 4).

**Fig. 5:**
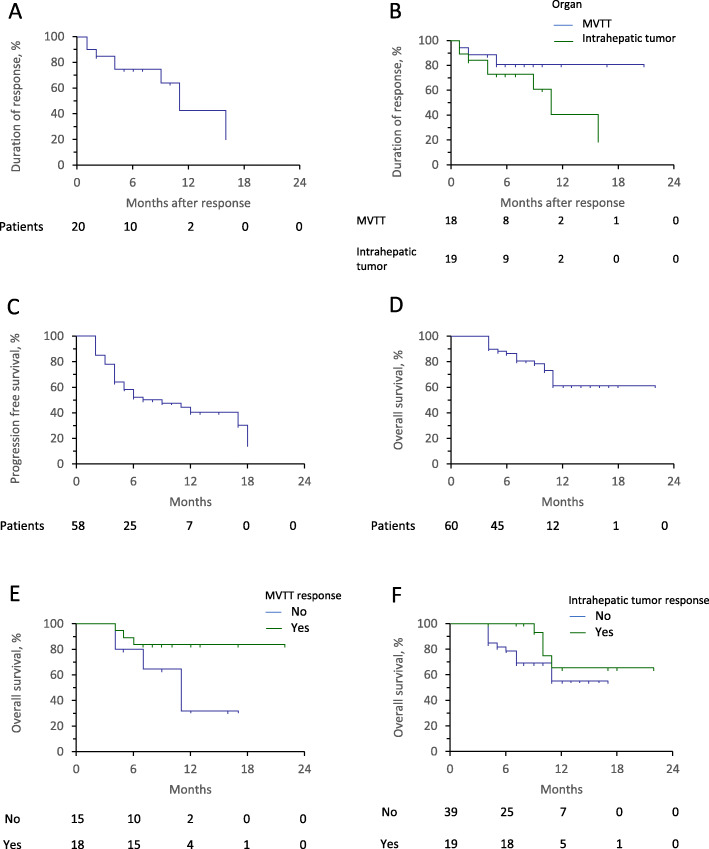
Survival analysis. **a** Duration of response in all 60 patients with advanced HCC who received first-line lenvatinib plus anti-PD-1 antibodies. **b** Duration of response in MVTT (*n* = 18; Note: these are the 18 patients who achieved a response to therapy) and intrahepatic tumors (*n* = 19) (*P* = 0.143). **c**, **d** Progression-free survival and overall survival in all 60 patients. **e** Overall survival among patients with MVTT and intrahepatic tumors (*n* = 33) who did and did not achieve a treatment response in MVTT (*P* = 0.037). **f** Overall survival among patients with intrahepatic tumors (*n* = 58) who did and did not achieve a response to treatment (*P* = 0.198)

The overall median DOR among all patients in the analysis was 10.5 months (95% CI, 6.9–14.1) (Fig. 5a). The median OSDOR for intrahepatic lesions was 10.5 months (95% CI, 6.8–14.2) and for MVTT was not reached (Fig. 5b; *P* = 0.143).

### Progressive disease analyses

Of the 10 patients with a best overall response of PD (INV assessment), eight had PD due to progression of intrahepatic disease, including one patient with an increase in tumor size, four patients with new lesions and three with both an increase in size of tumor and appearance of new lesions. Conversely, only two patients had PD due to the progression of extrahepatic disease.

Of the 50 patients who achieved disease control (CR, PR or SD), 15 experienced subsequent disease progression. Of them, 12 progressed because of intrahepatic tumor progression (two patients had enlargement of original tumors, five had appearance of new lesions and five had enlargement of original tumors and appearance of new lesions) and only three patients experienced PD due to extrahepatic lesions. This highlights that intrahepatic lesions contributed to most cases of PD.

### Patients’ survival

The overall median PFS among all patients was 7.0 months (95% CI, 1.7–12.3) and the overall median OS was not reached (Fig. 5c, d). In the 33 patients with MVTT, there was a significant difference in OS between those patients who did and did not achieve a treatment response in MVTT (Fig. 5e; *P* = 0.037). In the 58 patients with intrahepatic tumors, there was no statistically significant difference in OS among patents with and without a response to treatment (Fig. 5f; *P* = 0.198).

### Safety

In total, 38.3% of patients experienced at least one Grade 3 or 4 adverse event (Table 3). The most common were increased in gamma-glutamyl transferase (8.3%) and AST (8.3%), gastrointestinal bleeding (6.7%) and decreased white blood cell count (6.7%).

**Table 3:** Safety summary

	All patients (***N*** = 60)
≥1 Adverse event of Grade 3 or 4^a^, *n* (%)	23 (38.3)
Increased GGT	5 (8.3)
Increased AST	5 (8.3)
Gastrointestinal bleeding	4 (6.7)
Decrease in white blood cell count	4 (6.7)
Increased bilirubin	5 (8.3)
Decrease in neutrophil count	3 (5)
Ascites	3 (5)
Decrease in platelet count	1 (1.7)
Increased ALT	5 (8.3)
Hyponatremia	2 (3.3)
Pneumonia	1 (1.7)
Type I diabetes	1 (1.7)
Hypokalemia	1 (1.7)
Myocarditis	1 (1.7)
Hypophysitis	1 (1.7)
Bullous dermatitis	1 (1.7)
Hypertension	2 (3.3)
Grade 5^b^ adverse event, *n* (%)	1 (1.7)^b^

^a^Adverse events were graded according to the Common Terminology Criteria for Adverse Events v4.0; ^b^one patient whose main cause of death was immune hepatitis, and who died after treatment with high-dose hormone shock and liver protection

*ALT* alanine transaminase, *AST* aspartate transaminase, *GGT* Gamma-glutamyl transferase

## Discussion

The results of this study demonstrate differential organ-specific responses to combined lenvatinib plus anti-PD-1 monoclonal antibodies in patients with advanced HCC. There have been previous reports of organ-specific responses to immunotherapy in the second-line treatment of HCC [16, 17]. However, to our knowledge, this is the first report of organ-specific responses to first-line combination treatment with lenvatinib plus anti-PD-1 antibodies in patients with advanced HCC.

All patients in this study received first-line treatment for advanced HCC. Compared with a similar population of patients with advanced HCC receiving first-line therapy with lenvatinib plus pembrolizumab in the Phase Ib Keynote 524 study, a higher proportion of those in the present study were HBsAg positive (85 vs. 19%), had AFP ≥400 ng/mL (65 vs. 30%), BCLC Stage C disease (76.7 vs. 71%) and MVTT (50 vs. 30%) [25]. Our results show that first-line treatment with lenvatinib plus anti-PD-1 antibodies led to an ORR of 33.3%, a median DOR and PFS of 10.5 and 7.0 months and a median OS that was not reached. In comparison, patients in the Keynote 524 study achieved an ORR of 36% and a median DOR, PFS and OS of 12.6, 8.6 and 22 months, respectively [25]. These findings suggest that the combination of lenvatinib with a range of anti-PD-1 antibodies has a similar anti-tumor effect to lenvatinib plus pembrolizumab. In addition, the results of this study add evidence that combination therapy with tyrosine kinase inhibitors (TKIs) and immune checkpoint inhibitors is associated with a higher ORR and longer DOR than lenvatinib or pembrolizumab monotherapy [8].

Our study found a higher ORR and DOR in MVTT versus intrahepatic lesions. Furthermore, among 18 patients with a CR or PR in MVTT, six were able to achieve an R0 resection and, of these six patients, four were confirmed to have a pCR in MVTT. These findings provide evidence for the anti-tumor effectiveness of the combined lenvatinib plus anti-PD-1 antibody treatment strategy in HCC-related MVTT. Furthermore, of 33 patients in the present study with MVTT, 18 had differential tumor responses in intrahepatic tumors versus MVTT and among these patients 13 had a better tumor response for MVTT versus intrahepatic tumors. Although Kuo and colleagues previously reported higher ORRs for portal vein tumor thrombus versus intrahepatic lesions in patients receiving TKIs with or without immune checkpoint inhibitor therapy, patients in the present study received lenvatinib plus anti-PD-1 antibodies as a first-line treatment for advanced HCC, which is under investigation as a first-line therapy [16, 17]. In addition, in the present study, the OSRR in intrahepatic lesions and MVTT was 32.8 and 54.5%, which is slightly higher than reported by Kuo and colleagues among patients with advanced HCC receiving anti-PD-1 antibody monotherapy (intrahepatic: 14.7%; MVTT: 50%) [16]. Possible reasons for this include patients in the present study receiving first-line treatment only, compared with all comers in the Kuo study, and the relatively strong anti-tumor effect of combined lenvatinib plus anti-PD-1 antibodies compared with single or double agent immune therapy. In support of the latter explanation, recent clinical trials in advanced HCC have shown a trend for higher ORRs overall with combined TKI and anti-PD-1 antibody therapy [13, 25] versus anti-PD-1 antibody monotherapy [26] or dual immune-therapy strategies [27, 28]. Finally, in the present study, the OSRR for lung metastases was similar to previous reports of immune checkpoint inhibitor monotherapy (37 vs. 40% [16] and 41.2% [17]). However, due to the small number of patients with lung metastasis included in the present study, the conclusions that can be drawn are limited.

In our study, a total of 10 patients had a best overall response of PD (they did not achieve SD, PR or CR). Of these patients, eight were judged to have PD based on intrahepatic disease progression. Of these eight patients, seven had the appearance of new intrahepatic lesions. Among 15 patients who initially achieved disease control (SD, PR or CR) and subsequently progressed while on treatment, 12 had intrahepatic disease progression, of which 10 were due to the appearance of new lesions. These results suggest that, for patients with advanced HCC receiving combined TKI plus anti-PD-1 antibody treatment, disease progression is predominantly due to intrahepatic disease.

The differential OSRRs observed in our study have important implications for treatment decision making in patients with advanced HCC, particularly for the use of locoregional and surgical treatment. Our findings show that combined lenvatinib and anti-PD-1 antibody therapy leads to a higher OSRR and DOR in MVTT than in intrahepatic lesions. This suggests a proportion of patients with BCLC Stage C disease may have an opportunity to completely irradicate MVTT and be down-graded to BCLC Stage B or A. Such patients would then gain an opportunity to receive locoregional therapy or surgical resection [29]. In our study, patients who achieved a treatment response in MVTT had longer OS than those without a response. The presence of MVTT is known to be an important predictor of poor survival outcomes for patients with HCC receiving sorafenib [30]. However, compared with TKI monotherapy, combined TKI and anti-PD-1 antibody therapy can more effectively control or shrink MVTT and appears to offer better survival for patients [13, 25]. We propose that the longer survival times reported for such therapies is strongly related to control of MVTT. Therefore, careful evaluation of the response of MVTT to systemic therapy may improve the overall evaluation of response to treatment in patients with HCC.

The relatively low OSRR observed for intrahepatic tumors compared with MVTT in this study may be explained by the higher tumor burden associated with intrahepatic disease, which can prevent medication from entering tumors in this location, or could also be related to the immune function of the liver. Most cases of disease progression during treatment in the present study were due to the appearance of new intrahepatic lesions, highlighting the challenge of controlling intrahepatic disease during the treatment of HCC. Even patients with controlled intrahepatic disease may benefit from the concomitant use of locoregional (such as transarterial chemoembolization or radiotherapy) or surgical treatment to achieve comprehensive disease control. However, identifying effective combination treatments and optimal timing for adding surgical or locoregional therapies requires further investigation in controlled trials. Furthermore, the shorter DOR observed in the present study for intrahepatic lesions versus MVTT further highlights the potential benefit of the addition of surgical treatment or liver-directed therapy even for patients who achieve control of intrahepatic lesions during systemic therapy.

We recognize several limitations of this study. Firstly, the analysis included a relatively small number of patients which limits the strength of evidence and retrospective analyses have intrinsic limitations including delayed evaluation times and a lack of standardized processes compared with a prospective clinical trial. The data from the previously-reported controlled study may facilitate a more stringent analysis to re-examine the above findings. Secondly, there is currently no agreed standard for evaluating MVTT, and for this analysis we extended the RECIST v1.1 criteria to MVTT. The evaluation of MVTT response is a topic that requires further investigation in the future. Finally, while all patients received lenvatinib, anti-PD-1 monoclonal antibody treatment was not consistent for all patients, resulting in several different therapeutic combinations and the potential for therapeutic bias towards one combination over another. However, the ORR reported for currently available anti-PD-1 antibodies in HCC appear to be comparable both as monotherapy (within the range of 15–20% [11, 19, 26]) and when used in combination with vascular-targeted therapies (34–36% [14, 31, 32]). We therefore believe the phenomenon reported in this study is genuine, and warrants verification through the independent prospective investigation of fixed regimens.

In conclusion, in patients with advanced HCC, first-line treatment with lenvatinib plus anti-PD-1 antibodies resulted in a better tumor response in MVTT compared to intrahepatic lesions. We propose that complete MVTT necrosis can lead to downstaging and subsequent eligibility for surgical liver resection in a proportion of patients with advanced HCC. This combination treatment strategy may therefore allow selected patients with MVTT at HCC diagnosis to access surgical and locoregional treatments, with the potential for increased long-term survival versus current treatment options.

## Data Availability

The datasets used and/or analysed during the current study are available from the corresponding author on reasonable request.

## Abbreviations

Anti-PD-1: Anti-programmed cell death-1
BCLC: Barcelona Clinic Liver Cancer
CNLC: China National Liver Cancer
CR: Complete response
DCR: Disease control rate
DOR: Duration of response
ECOG: Eastern Cooperative Oncology Group
HBsAg: Hepatitis B surface antigen
HCC: Hepatocellular carcinoma
IIR: Independent imaging review
INV: Investigator
MVTT: Macrovascular tumor thrombi
ORR: Objective response rate
OS: Overall survival
OSDCR: Organ specific disease control rate
OSRR: Organ-specific response rate
pCR: Pathological complete response
PFS: Progression-free survival
PD: Progressive disease
PR: Partial response
RECIST: Response Evaluation Criteria in Solid Tumors
SD: Stable disease
TKI: Tyrosine kinase inhibitors
